# Evolutionary Perspectives on the Developing Skeleton and Implications for Lifelong Health

**DOI:** 10.3389/fendo.2020.00099

**Published:** 2020-03-04

**Authors:** Alexandra E. Kralick, Babette S. Zemel

**Affiliations:** ^1^Department of Anthropology, University of Pennsylvania, Philadelphia, PA, United States; ^2^Division of Gastroenterology, Hepatology and Nutrition, The Children's Hospital of Philadelphia, Philadelphia, PA, United States; ^3^Department of Pediatrics, The University of Pennsylvania Perelman School of Medicine, Philadelphia, PA, United States

**Keywords:** osteoporosis, evolution, growth, skeleton, nutrition, physical activity, longevity

## Abstract

Osteoporosis is a significant cause of morbidity and mortality in contemporary populations. This common disease of aging results from a state of bone fragility that occurs with low bone mass and loss of bone quality. Osteoporosis is thought to have origins in childhood. During growth and development, there are rapid gains in bone dimensions, mass, and strength. Peak bone mass is attained in young adulthood, well after the cessation of linear growth, and is a major determinant of osteoporosis later in life. Here we discuss the evolutionary implications of osteoporosis as a disease with developmental origins that is shaped by the interaction among genes, behavior, health status, and the environment during the attainment of peak bone mass. Studies of contemporary populations show that growth, body composition, sexual maturation, physical activity, nutritional status, and dietary intake are determinants of childhood bone accretion, and provide context for interpreting bone strength and osteoporosis in skeletal populations. Studies of skeletal populations demonstrate the role of subsistence strategies, social context, and occupation in the development of skeletal strength. Comparisons of contemporary living populations and archeological skeletal populations suggest declines in bone density and strength that have been occurring since the Pleistocene. Aspects of western lifestyles carry implications for optimal peak bone mass attainment and lifelong skeletal health, from increased longevity to circumstances during development such as obesity and sedentism. In light of these considerations, osteoporosis is a disease of contemporary human evolution and evolutionary perspectives provide a key lens for interpreting the changing global patterns of osteoporosis in human health.

## Introduction

Globally, musculoskeletal disorders are one of the five leading causes of years lived with disability, affecting an estimated 1,270,630,000 people (1). While the contribution of osteoporosis to this global statistic is uncertain, the magnitude of the burden is reflected by the fact that in the U.S., osteoporosis-related fractures are responsible for more hospitalizations than heart attacks, strokes and breast cancer combined (2). Osteoporosis is primarily a disease of aging whereby age-related losses in the mass and structural properties of bone lead to increased bone fragility and risk of fracture (3, 4). Clinically, osteoporosis in adults is defined as a bone mineral density measurement at least 2.5 standard deviations below the mean at the spine, femoral neck or total hip for young, healthy adults (5), or the occurrence of an osteoporotic (low-trauma) fracture of the hip, vertebra, proximal humerus, pelvis, and some wrist fractures in the context of low bone density (1–2.5 standard deviations below the mean) (6).

**Figure 1:**
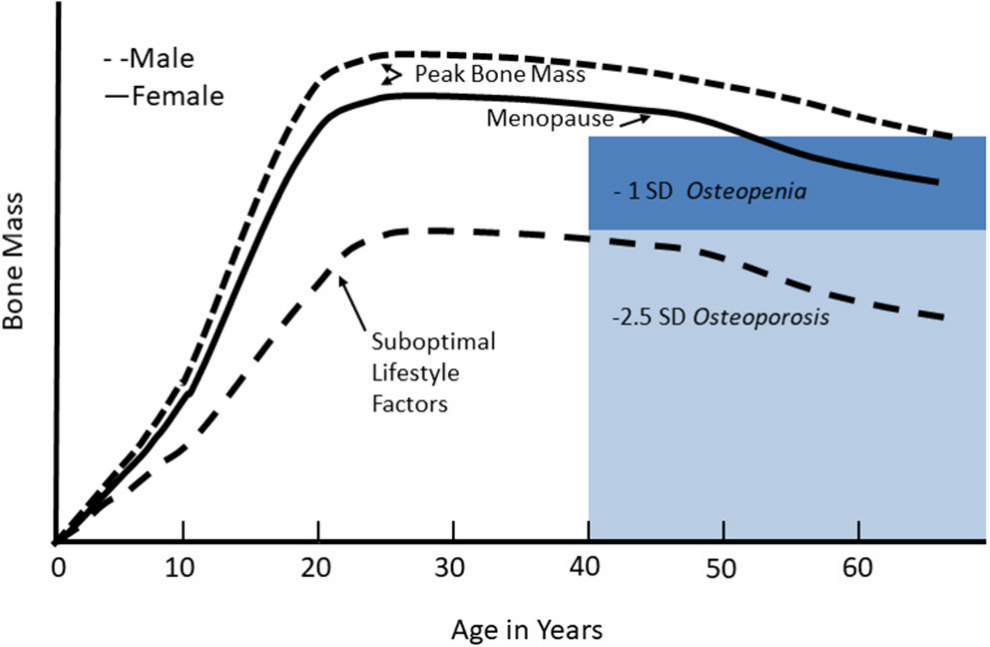
Changes in bone mineral mass across the life cycle. Bone mineral mass increases during growth and reaches a plateau, referred to as peak bone mass, in young adulthood. Women lose bone rapidly in the first few years of the menopausal transition, and then both men and women continue to lose bone gradually in older age. For adults, low bone mass, or osteopenia, is defined as 1–2.5 standard deviations below peak bone mass; osteoporosis is defined as bone mass <2.5 standard deviations below peak bone mass. With suboptimal lifestyle factors, failure to achieve optimal peak bone mass reduces the age of onset of osteopenia or osteoporosis given the usual age-related bone mass. Reproduced from Weaver et al. (7) under the Creative Commons CC-BY License.

Although osteoporosis is primarily a disease of aging, it is thought to have origins in childhood. The bone mineral content of the body increases as the size of the skeleton expands during growth (Figure 1). During the second to third decades, gains in bone mineral content and density reach a plateau, referred to as peak bone mass. This process has a strong genetic component, but is also sensitive to the physiological milieu and behaviors that can influence bone accretion and result in suboptimal peak bone mass. Peak bone mass is a strong predictor of osteoporosis in later life. Because childhood and adolescence are periods of rapid bone accrual leading up to peak bone mass, they are believed to be critical for optimizing peak bone mass and preventing or delaying the onset of osteoporosis in older age.

Here we will describe the relationship of growth, body composition, maturation, and behaviors to peak bone mass attainment in contemporary populations, what is known from skeletal populations, and the evolutionary implications of skeletal development and osteoporosis.

## Development of Peak Bone Mass and Peak Bone Strength

**Figure 2:**
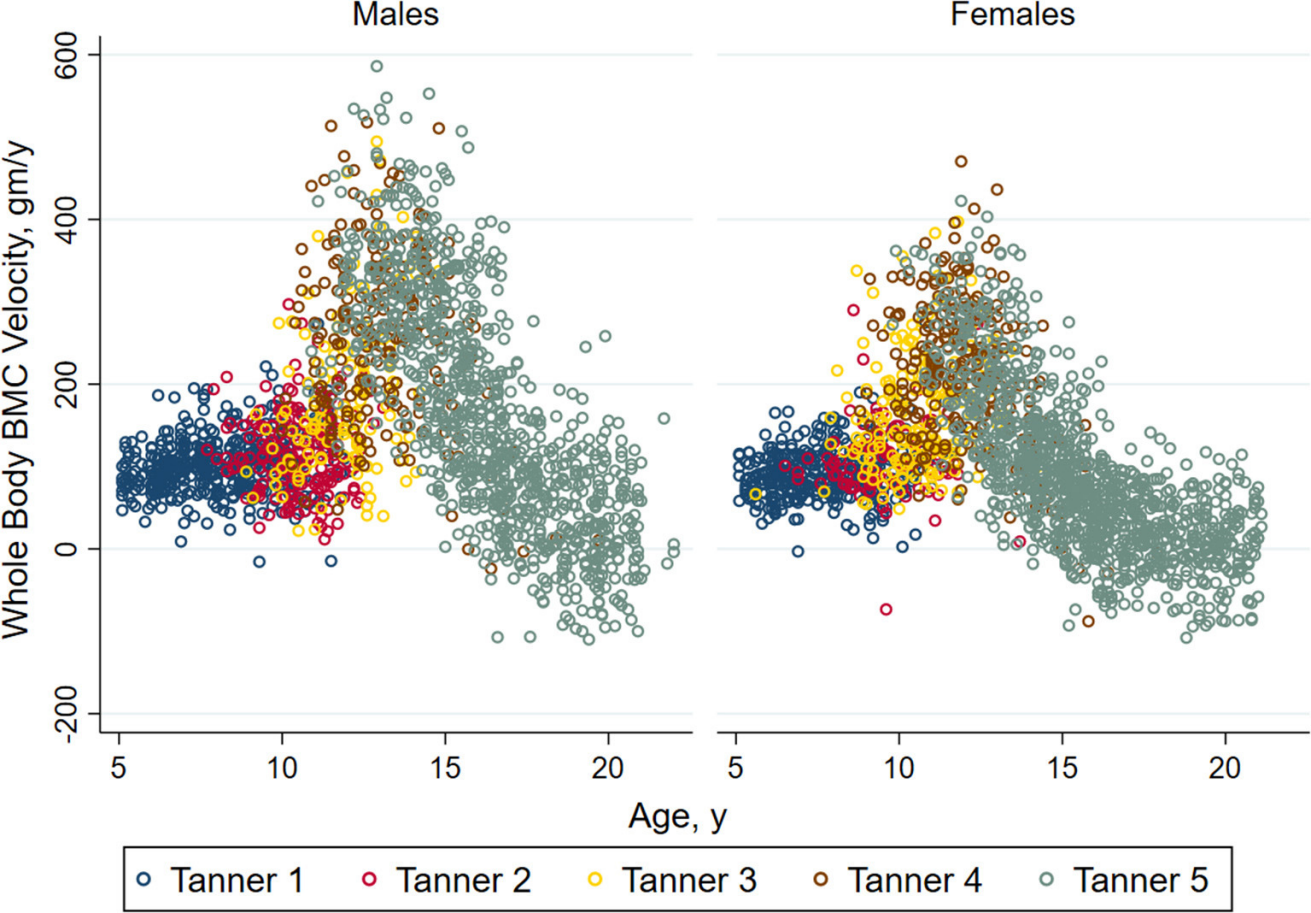
Annual velocity (bone accrual) in whole body bone mineral mass in healthy non-African American children and adolescents according to Tanner stage of sexual maturation (breast stage for girls and testicular volume for boys). Individual values for bone accrual are shown relative to age with different colors representing the stages of sexual maturation. For both males and females, children in Tanner stages 1 and 2 have similar whole body bone accrual. Annual velocity is highest in Tanner stages 4 and 5, and declines with age among children in Tanner stage 5. Reproduced from Kelly et al. (8) with permission.

Bone accrual and the development of peak bone mass occurs through the delicately coordinated actions of bone deposition and resorption that are sensitive to genetics, hormones, mechanical loading through physical activity, other behaviorally mediated factors (e.g., diet), and insults from the internal (e.g., inflammatory cytokines), and external environments (e.g., low sunlight exposure) (7). During childhood, the rate of bone accrual is relatively constant, but changes as puberty progresses. As shown in Figure 2, prepubertal children (Tanner breast/genital stage 1) and those in early puberty (Tanner stage 2) show similar rates of bone accrual. In mid-puberty, i.e., Tanner stages 3 and 4, the rate of bone accrual increases markedly, and even in the later stages of puberty (Tanner stage 5), bone accrual rates are still at their peak for some youth. The rate of bone accrual reaches a maximum 6 months to 2 years after peak height velocity, depending on the skeletal site examined (9). Approximately 33% of adult total bone mass is accrued in the 2 years before and 2 years following peak height velocity. Bone accrual continues after cessation of linear growth; in fact, 7–11% of adult bone mass is gained after the cessation of linear growth (9). Bone accrual is completed and peak bone mass attained earlier in females than in males (9, 10), although the exact timing at the individual level is uncertain.

Peak bone mass could be the single most important factor to prevent osteoporosis later in life (11, 12). Two lines of evidence support this hypothesis. First, the variability in bone mass and density at the time of peak bone mass is large compared to age-related bone loss in older adulthood. So, attaining a higher peak bone mass mitigates against age-related bone loss to a level that poses risk for fracture (13, 14). Secondly, a computer simulation study of bone remodeling showed that at the population level, shifts in peak bone mass have a greater impact on delaying the onset of osteoporosis than shifting the age at menopause, a major cause of age-related bone loss in women. Indeed, this simulation showed that an increase of 10% in the magnitude of peak bone mass can delay the onset of osteoporosis by 13 years for much of the population (12).

Bone strength is determined, in part, by bone mineral mass and density. The structural properties of bone also contribute to bone strength. Characteristics such as the thickness, density and porosity of cortical bone, and trabecular microarchitecture (trabecular thickness, number, and spacing), together determine the structural strength of bone. It is unknown whether peak bone strength occurs at the same time as peak bone mass attainment. Recent studies suggest redistribution between the trabecular and cortical bone compartments after the age at which peak bone mass is attained. One of the first studies using high-resolution peripheral quantitative computed tomography (a technology that can quantify the properties of trabecular and cortical bone) demonstrated fairly rapid trabecular bone density loss in the tibia, radius and spine in females and to a lesser degree in males after peak bone mass attainment. However, increases in cortical bone occurred through the 3rd decade. The increases in cortical bone density into the third decade have been confirmed in other studies, with inconsistent results for trabecular bone density (15–17).

## Factors that Influence Development of Peak Bone Mass and Strength

The development of peak bone mass during adolescence is influenced by heredity, growth, sexual maturation, physical activity, diet, nutritional status, and other behaviors such as sleep, and overall health. These factors have important implications for understanding temporal and geographic variation in osteoporosis.

### Heredity and Genetics

Differences between some population ancestry groups in bone density distributions are present during both childhood and adulthood. Areal bone mineral density is greatest for African Americans, and Europeans have higher areal bone mineral density than Asians and Hispanics (18–20); these differences are thought to be due to differences in genetic potential for peak bone mass (21). The population ancestry differences are also mirrored by differences in fracture rates among both children and adults (22, 23). For example, in the Women's Health Initiative study, African American women had a 49% lower risk of fracture than white women (22) similar to other reports (24–27), Asian populations also have a lower incidence of hip fractures than white US populations (28–30). During development of peak bone strength, African Americans have greater maturation-specific trabecular density and cortical structural strength (31–33). The evolutionary basis for these population ancestry differences in bone density and strength are unknown.

Familial studies show that ~60–80% of osteoporosis risk is attributed to heredity (11, 34). Familial concordance is strong (35, 36), and is expressed prior to puberty (37). More recently, genome wide association studies in adults have discovered more than 60 loci associated with bone density (38–41) and 14 loci associated with fracture risk (41). An additional 518 loci have been associated with ultrasound heel estimated bone mineral density by heel ultrasound (42, 43). Genetic risk scores, calculated as a tally of the number of risk alleles at these loci, associate with bone density during childhood (21, 44–47). Combined, these many loci only explain about 20 percent of the variability in bone density and related outcomes (43), so a large portion of the estimated heritability of low bone density remains to be identified.

### Growth and Maturation

**Figure 3:**
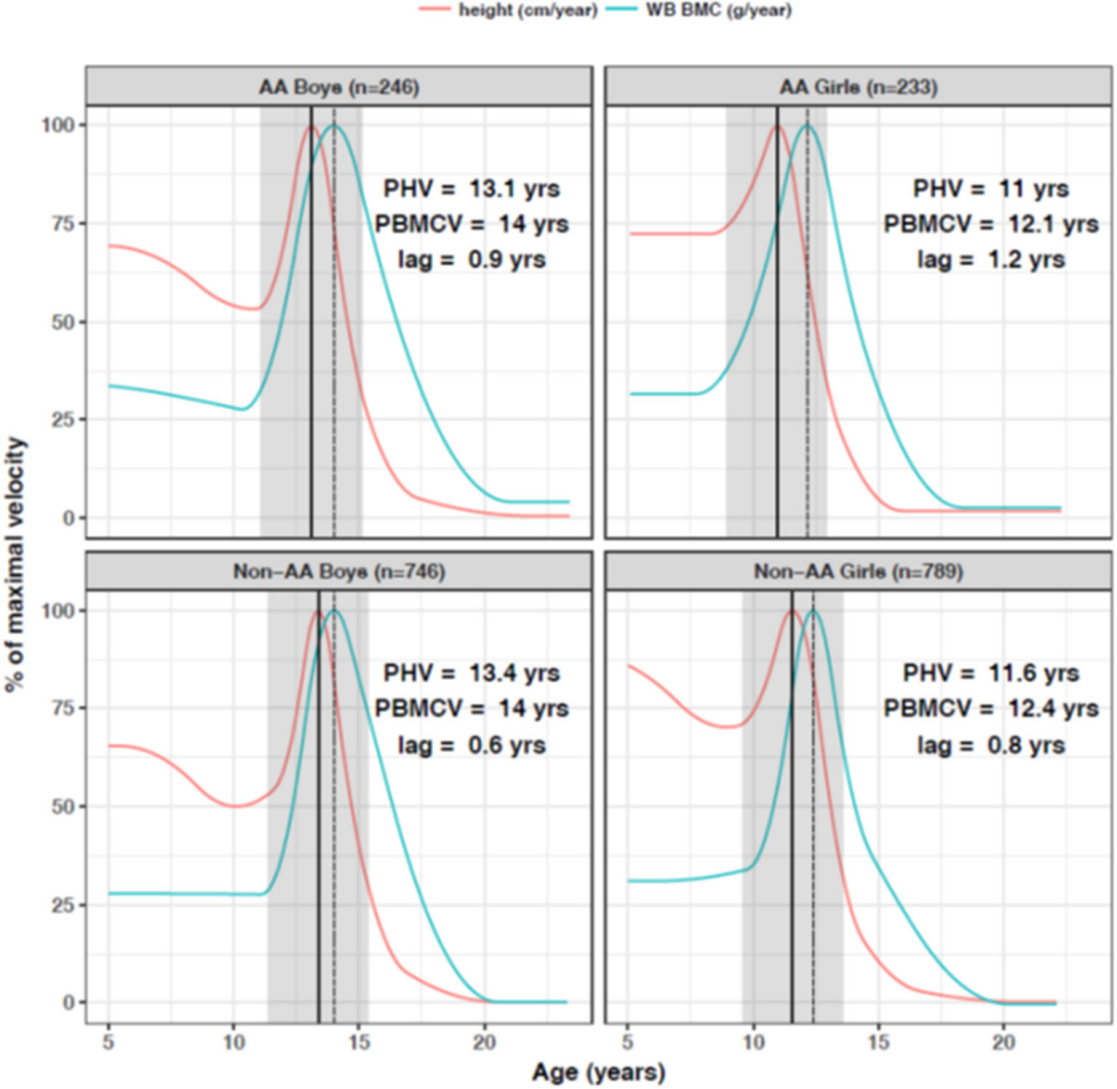
Peak bone accretion occurs after the pubertal growth spurt in height in African American (AA) and non-African American (non-AA) boys and girls. In the 2 years before and 2 years after the growth spurt in height, children gain about 33% of adult total bone mass. Bone accrual continues after cessation of linear growth. Reproduced from McCormack et al. (9) with permission.

The bone mineral content of the total body and subregions increases along with skeleton size during growth and maturation. Most pediatric studies have used dual energy x-ray absorptiometry (DXA) to measure bone mineral content and density and the association with height in growing children is very strong. More importantly, for children of the same age, those who are taller for age have greater bone mineral content and areal bone mineral density (48). Figure 3 illustrates the rate of bone accretion relative to the timing of the pubertal growth spurt in height (9). The maximum rate of bone accretion is preceded by peak height velocity which occurs 6 months to 2 years depending on the skeletal site. Cortical dimensions, such as periosteal circumference and cortical thickness also increase profoundly during growth, and scale to the length of growing long bones in order to sustain structural competency (33).

As described above, pubertal maturation has a profound effect on bone accretion. The rate of bone accretion in childhood and early puberty is fairly constant. By puberty stage 3, defined breast development in girls and genital development in boys (49), the rate of bone accrual for the whole body, spine and hip begins to increase and is greatest in stages 4 and 5 (8). At the end of puberty, male cortical thickness is greater than in females (50). While sex differences in bone mass are small prior to puberty, it is the sex differences in pubertal gains in bone mass, dimensions and strength that establish the sex-based differences in osteoporosis and fracture later in life. Osteoporosis disproportionally affects women, who have a two-fold greater lifetime risk of bone fracture (51).

Pubertal timing has long lasting effects on bone mineral content and density. One longitudinal study of prepubertal girls followed to young adulthood showed that earlier maturing girls had greater bone density prior to menarche and into adulthood (52). A population based cohort also found later age at maturation associated with lower bone density in both males and females in young adulthood (53), but another study suggested that later-maturing males eventually catch up to their earlier maturing peers (54). A genetic study demonstrated that genetic risk scores based on variants associated with later pubertal timing in boys and girls were associated with lower bone mineral density, supporting a causal relationship between later puberty and osteoporosis risk in both sexes (47). The detrimental effect of later pubertal timing on bone accrual in women appears to be sustained well into adulthood (52).

### Physical Activity

Physical activity is perhaps the single most important lifestyle factor influencing peak bone mass. Bone responds to loads and mechanical use resulting from physical activity. This phenomenon was first described as Wolff's law (55), which proposed that bone morphology adapts to mechanical forces from muscle load (56, 57). Building upon this law, the “mechanostat theory” developed by Frost explained how bones adapt their strength to the mechanical loads to which they are exposed (58). Bone turnover occurs throughout the lifecycle to maintain healthy bone through the delicately coordinated action of osteoblasts and osteoclasts which control bone formation and resorption. When bone formation exceeds bone resorption, bone modeling occurs. During longitudinal bone growth, mechanical stimulation increases the bone modeling process to reshape bone in a manner that minimizes risk of fracture (58). Mechanical stimulation from high magnitude strains produces more bone modeling than lower strains at higher frequency. Modeling is less effective after skeletal maturity. The relationship between mechanical loads and cortical development was best documented in tennis players. Professional tennis players show marked asymmetry due to about 20% more bone mineral content and muscle mass in the dominant arm (59–61). The benefit of playing tennis or squash on the bones of the playing arm was approximately doubled for females who started playing at or before menarche compared to those who began training at a later age, demonstrating the increased responsiveness of bones to adapt to loading forces earlier during development (60). Numerous studies have confirmed the effects of physical activity on bone mineral content and density, dimensions and strength during childhood and adolescence for those engaged in other sports, such as gymnastics (62), as well as non-competitive weightbearing physical activity (46, 63, 64). Even in young adulthood, the benefits of weight-bearing physical activity on increasing or sustaining bone strength have been documented (65–67).

Several studies suggest that the increased bone mass and strength derived from physical activity in childhood may not be sustained at the same level (bone loss can occur) if physical activity levels decline. However, the advantage in bone mass and strength remains compared to those who never engaged in high levels of physical activity during growth. This has been demonstrated, for example, in studies of female soccer players (68), retired gymnasts (69), and former weightlifters (70).

### Diet and Nutritional Status

There are several dietary constituents that are essential for bone health. The mineral matrix of bones is made of hydroxyapatite, a compound of calcium and phosphate. Bones are the primary reservoir for calcium, an essential nutrient that is tightly regulated in the blood to sustain important physiologic functions such as nerve conduction. Vitamin D is needed for calcium homeostasis. Thus, calcium and vitamin D are among the most important nutrients for sustaining bone mineral accrual and optimizing peak bone mass. Young children are particularly vulnerable to nutritional rickets from either calcium or vitamin D deficiency (71). Some randomized control trials of calcium and/or vitamin D support the importance of these nutrients for optimizing bone accretion during childhood (7). The importance of these nutrients during the most rapid phase of bone accretion during adolescence is uncertain (72). The effects of very low calcium intake during childhood may be long lasting; women who reported consuming less than one serving of milk per week during childhood and adolescence had lower hip bone mineral density compared to those who consumed more milk, and fracture risk was greater in those with low milk consumption (73).

Other nutrients are also important for bone accretion based, including but not limited to copper, zinc, magnesium, vitamin C and K, and protein (74). The effects of these individual nutrients on bone accretion during growth and development in humans has been difficult to discern in the absence of frank deficiency states since many of these nutrients are combined in overall dietary patterns. For example, higher fruit and vegetable consumption is associated with greater bone accretion in young children (75) and adolescents (76). A supplementation study using a prebiotic inulin type of dietary fiber showed increased calcium absorption and greater gains in bone mineral content in peripubertal children (77). A “prudent/healthy” dietary pattern (high intakes of vegetables, fruits, low-fat milk and dairy products, whole grains, fish, beans, and nuts) was associated with lower risk of fracture in adolescents (78). Thus, while diet is clearly an important factor, the role of many individual dietary constituents has not been fully delineated, but diets rich in fruit and vegetables with adequate calcium intake will support optimal bone accretion.

Nutritional status in terms of extremes of underweight and overweight have adverse consequences for bone accretion and development of optimal bone strength. Adolescents with anorexia have low bone density and reduced strength compared to normal weight peers, and these effects can be long lasting (79, 80). At the other end of the spectrum, children with obesity have greater bone mineral content and density and greater cortical thickness than non-obese children (81). However, they experience greater risk of fracture (82) and greater complications from fractures (83) than peers who are not obese.

In sum, genetic differences between and within populations suggest that selective pressures may have influenced the development of bone fragility in some populations. However, the nature of these evolutionary forces remains unknown. Developmental patterns of growth and maturation, especially timing of sexual maturation, have long lasting effects on bone health through the life cycle. Lifestyle factors such as physical activity patterns, diet, and nutritional status also influence bone mineral accretion and the development of peak bone mass. The implications through human history are considered below.

## Development of Peak Bone Mass and Strength in Skeletal Populations

From early human history to today, changes in subsistence strategy, physical activity pattern, and occupation have been inferred in archaeological skeletal collections using the relationships between physical activity patterns and musculoskeletal stress markers, cross-sectional bone properties, bone mass, and skeletal strength. While there is some caution to these interpretations because of the osteological paradox, i.e., the uncertainty in inferring cause of death in skeletal remains because most skeletal markers of disease require prolonged disease duration, these conclusions have implications for the interpretation of osteoporosis as a disease of modern human evolution.

Differences between skeletal collections in bone mass and strength are not uniform across all skeletal sites due to activities patterns and resulting mechanical loading at each skeletal site (84). Most skeletal research is represented by adult samples, but a few skeletal collections include infants and children. Childhood activity patterns are inferred from adult characteristics based on the greater responsiveness to mechanical loading of bone during childhood, and the need to sustain physical activity throughout life to maintain bone's structural competency. The relationship between skeletal features and biomechanical stimuli is used to reconstruct past lifeways from skeletal collections (85).

### Musculoskeletal Stress Markers

Subsistence strategy, occupation, and socioeconomic status for past peoples have been linked to musculoskeletal stress markers (enthesiopathies) based on interpretations of the effects physical activity on bone (86, 87). The enthesis is the area where a tendon or ligament attaches to bone, so enthesopathies, also known as a musculoskeletal stress markers, are changes to that region which are assumed to reflect changes in the attaching musculature (88). Studies of musculoskeletal stress markers and habitual activities began in the 1950s, but received greater attention in the 1980s (89). A meta-analysis of these studies concluded that agriculturalists had the lowest entheseal changes in the upper body, followed by hunter gatherers, and then by those working in industry, for both males and females. These findings suggest that individuals from industrialized populations were not as adequately adapted to adult workloads as were individuals from other subsistence groups, although age-related differences between populations were unknown (87). Confounding factors in studies that use entheseal changes to interpret effects of habitual physical activity on bone include the difference in responsiveness to loading forces in growing vs. older bone (56); the difference between fibrous and fibrocartilaginous entheses (88), and age-related changes (90, 91). More robust methods of interpreting physical activity from skeletal collections are based on cross-sectional area and geometry of long bones using imaging technologies such as x-ray, MRI, and CT scanning.

### Cross-Sectional Bone Geometry

Studies of cross-sectional bone geometry began in the late 1970s (89). Here we offer some examples describing characteristics of bone strength at a number of skeletal sites. Ruff compared geometric properties of the femoral midshaft (such as cross-sectional area, polar moment of area, etc.) in pre-agricultural and agricultural young adult skeletons (both males and female) from the Georgia coast. The agricultural sample had significantly lower values for nearly every geometric property measured, such as cortical area, medullary area, and polar second moment of area in the midshaft and subtrochanteric regions. To some degree, this was due to smaller bone length in the agricultural sample, but after adjusting for the smaller bone length in the agricultural samples, numerous differences remained significant especially in subtrochanteric cross-sectional properties. Results were more pronounced for females. These findings are consistent with a decline in mechanical loading associated with the transition to agriculture (92). A subsequent study of more than 1,800 specimens across Europe, from the Upper Paleolithic (11,000–33,000 years BP) to the twentieth century showed a large decline in anteroposterior bending strength in the femur and tibia that began during the Neolithic period (~4,000–7,000 years BP), continued through the Roman period (~2,000 years BP), and then stabilized (93). They found little change in humeral strength measures. Declining lower limb strength appears to be due to lower mobility (distance and speed of travel, and roughness of terrain) and increasing sedentism, which was gradual over time with the transition to sedentism and agriculture, and has not changed substantially with industrialization.

The upper body is subject to different lifestyle factors than the lower body. Differences in the cross-sectional structure of the humerus midshaft were examined in a skeletal collection from medieval York England (eleventh to sixteenth centuries) (94). The sample compared remains of lay benefactors (both males and females) from a church burial ground to later remains of brethren from when the church became a priory. Asymmetry in the polar moment of area between the right and left humeri was measured as an indicator of habitual loading of one limb compare to the other, such as would occur with iron working, carpentry or stone working. Monastic and lay males did not differ in the magnitude of asymmetry between right and left humeri, but both groups of males had significantly greater asymmetry compared to females. This is consistent with documented sex-specific patterns of participation in very different trades and habitual activities. However, average bone strength from polar moment of area values were greater for lay males than monastic males, which was likely a result of overall lesser physical activity levels of the monk brethren (94).

Vertebrae from Swedish and English medieval archaeological samples were compared to clinical samples in Sweden, showing an increase in vertebral height and reduction of vertebral width and vertebral cross-sectional area from medieval to post-medieval to modern time. The secular trend for increased stature in Europe over this time period accounted for increased vertebral height. The reduced vertebral width likely reflects declining physical activity in childhood and adolescence coincident with the increase in technological development and decreased strenuous physical activity (95).

Few studies have included children. Neolithic and Byzantine era samples of adults and children from Turkey were compared to contemporary data from the Denver Growth Study. Both Turkish skeletal collections had larger cortical and total areas in their femora than American urban adults, and these differences were established by age 6 years (96). These results emphasize the importance of physical activity patterns established in childhood for lifelong skeletal strength.

Lastly, a major study compared trabecular microstructure of the proximal femur by microcomputed tomography from 32 non-human primate species and archeological collections of mobile foragers (~5,000–7,000 years BP) and sedentary agriculturalists (~700–860 years BP) from North America. The forager population had significantly higher bone volume fraction, thicker trabeculae, and lower relative bone surface area in the proximal femur compared to the agriculturalists, and were similar to other primate species relative to estimated body mass (97). However, the agricultural specimens had the lowest bone volume fraction (bone volume to total volume) and thinnest trabeculae across species. These findings provide evidence of the effects of subsistence strategy on trabecular microarchitecture that is likely due to differences in physical activity levels, although diet may also be a contributing factor.

Overall, these studies demonstrate that declining physical activity levels attributable to sedentism and agricultural practices resulted in lower cortical dimensions and strength, and less favorable trabecular microstructure in lower limbs (97–101). Upper limb bone strength is influenced by activity patterns that are more occupation specific, rather than dependent on larger trends in recent human evolutionary history. The limited evidence from skeletal collections that include children support contemporary studies showing that the developing skeleton is responsive to highly mechanical adaptation, with differences in skeletal populations emerging at a young age.

### Evidence of Osteoporosis and Fractures in Skeletal Collections

Evidence of osteoporosis and age-related bone loss in archeological skeletal collections is limited. Awareness of osteoporosis dates back at least to the mid eighteenth century as it was first described in 1751 by Joseph Guichard Duverney (102). A historic population from 1700 to 1850 from London showed patterns of age-related trabecular bone loss in vertebral bodies similar to that of contemporary populations (103). Indeed, vertebral crush fractures are the most commonly reported osteoporotic fracture found in archaeological material, although wrist and hip fractures are documented occasionally (104). Age-related cortical bone loss has been reported; in a skeletal collection from Nubia dating between 350 BC and 1400 AD, the femoral cortical thickness significantly declined with age in females but not in males, and the decline in females began earlier than in modern females (105). In a sample of British medieval adult skeletons age-dependent cortical bone loss was broadly similar to modern Europeans, particularly for post-menopausal women, using metacarpal radiogrammetry (106). Moreover, low metacarpal cortical index was significantly associated with rib and vertebral crush fractures, but hip and wrist fractures were rare. In sum, patterns of bone loss were similar between these medieval women and contemporary populations, but the nature of osteoporotic fractures differed.

Determining elements of lifestyle that might contribute to osteoporosis from archeological samples is challenging. A study of osteoporosis among ancient Egyptians of different social classes in the Old Kingdom of Giza offers some insight. Bone density by DXA and microarchitecture by scanning electron microscopy were used to examine the radius, femoral head, and fourth lumbar vertebra. Rates of osteoporosis varied by occupation and sex. Overall, bone density was lower in females than in males. Among males, osteoporosis was more frequent in workers than in high officials, whereas in females, osteoporosis was more prevalent in high officials compared to workers. This may have been a result of higher workload and nutritional stress for male workers compared to male high officials and a more sedentary lifestyle for female high officials compared to female workers (107).

Low bone mass is reported in bioarcheology studies of historic skeletal collections, but evidence of osteoporotic fracture itself is uncommon (108). However, evidence of their incidence in the archaeological record is growing (109). It is important to consider that most skeletal populations do not have known age and that age determination of older skeletons comes with challenges (106).

### The Osteological Paradox

Interpreting the health of skeletal population requires consideration of the osteological paradox. The osteological paradox refers to the problems in reconstructing characteristics of once alive people from those who died (110). Three key issues that complicate attempts to evaluate the health of past human populations using archaeological skeletons: (1) demographic non-stationarity, (2) selective mortality, and (3) hidden heterogeneity in risk. In terms of osteoporosis, those who showed evidence of severe osteoporosis and osteoporotic fracture were those who survived with the disease for a period of time. There may have been individuals with the disease that died at an earlier age. We must consider what conditions were survived and which may have caused death and other factors related to individual mortality when attempting to interpret the incidence of a historic disease based on the individuals in a population who died. This is very different from contemporary methods for diagnosing osteoporosis in living populations making it difficult to compare deceased skeletal populations with those living today.

## Evolutionary Implications

The human skeleton is gracile compared to earlier hominin fossil skeletal evidence as well as living great ape skeletons. A number of evolutionary explanations have been proposed to explain this trend. First, past populations show skeletal evidence of higher physical activity levels (93, 111). Prehistoric bronze age agriculturalist women had tibial rigidity exceeding that of living modern athletes in Europe, and Neolithic men had similar tibial rigidity and shape ratios to that of modern cross-country runners (112). In lower limbs, declining bone dimensions, density and strength were not evident in human populations until the transition from hunting and gathering to food production and sedentism in the Neolithic around 10,000–12,000 years ago (113). In other words, the agricultural transition signaled changes in the mechanical forces that shape the human skeleton.

From the archeological record, it is difficult to estimate the effect of lesser bone dimensions, density and strength on prevalence of osteoporosis, mainly because of the osteological paradox. Evidence of osteoporosis from skeletal and historic populations exists, but osteoporotic fracture mainly manifests as vertebral crush fractures rather than osteoporotic fractures of the wrist and proximal femur. As simulations have shown, at the population level small increments in peak bone mass and strength can profoundly delay the onset of osteoporosis. Maintaining physical activity through adulthood also prevents or delays the onset of age-related declines in bone density. Physical activity levels in modern people, and in particular children for whom responsiveness to physical activity is greatest, may be reaching an unprecedented low, and is likely the primary reason for increasing cases of osteoporotic fracture (111) and the contemporary pattern of more devastating, life-altering fractures of the wrist and hip.

The transition to agriculture also brought dietary changes. Reconstructed paleolithic diets relied on varied resources, containing larger amounts and types of fruits, vegetables, nuts, seeds, tubers, and fish/game (114–116). This diet differed in fiber content, micronutrient and antioxidant capacity compared to contemporary diets, and would have more favorably supported bone health as suggested by current studies of diet and bone health described above (7).

Another consideration for the propensity for osteoporosis in modern people is the evolution of human longevity. Humans live the longest of any primate and are the longest living mammal (117). In the 1970s, the maximum human lifespan was 113 years while the maximum chimpanzee lifespan in ideal zoo conditions was 55 years (118). Maximum human lifespan was close to 95 years in medieval England, in classic Rome and Greece, in the Neolithic, and even in the Mesolithic and upper paleolithic (118). However, a sizeable portion of the population living into old age is a relatively recent change as early as the nineteenth and twentieth centuries (118).

One possible explanation for the evolution of increased human longevity is the role that grandmothers play in caring for grandchildren (119). Post-menopausal women, the demographic group most affected by osteoporosis, have completed their reproductive life and no longer contribute to the gene pool by bearing more children. However, they have great potential to contribute significantly to the survival of their progeny, so their evolutionary significance continues. Provisioning of food and childcare by grandmothers in a non-reproductive period of life favors longer lives and greater survival over generations (120). Maternal grandmothers improve the nutritional status of children and survival probabilities in rural Gambia (121). Among the Hadza hunter gatherers, grandmothers spend the most time foraging when the grandchildren are receiving the least from mothers, and they forage least when the grandchildren receive the most from mothers (122). Following a lifetime of high physical activity in hunter-gatherer subsistence, these grandmothers continue to engage in physical activity to provision for their families thereby continuing to maintain skeletal bone strength. Extended human longevity, particularly for women, speaks to the importance of continued physical activity to delay the onset of bone fragility.

## Conclusion

The developing skeleton is highly responsive to lifestyle patterns. Peak bone mass and strength are major determinants of bone fragility later in life and are shaped during childhood and adolescence. Growth, timing of pubertal maturation, physical activity and diet are among the factors that influence the magnitude of peak bone mass. In the context of adequate health and nutrition, physical activity is the most important *modifiable* factor promoting lifelong bone strength. Studies of skeletal populations demonstrate declines in skeletal robusticity, cortical dimensions, and trabecular microarchitecture in association with changing subsistence strategies and accompanying lifestyle changes. These changes in subsistence strategy constituted a shift for many human populations from foraging or hunting and gathering for sustenance to food production and agriculture. The activity of hunting and gathering involved obtaining sustenance from the collection and/or hunting of a wide variety of wild foods that provided adequate nutrients to support a robust skeletal phenotype. While there are still a number of hunter gatherers today, most foragers began using some cultivation strategies around 13,000 years ago and eventually started using agriculture. These different subsistence strategies entail different activity patterns, with agriculture typically characterized by more sedentary lifestyles. The confluence of increased longevity and reduced physical activity throughout the lifecycle exacerbate the problem of osteoporosis. As such, osteoporosis is a disease of contemporary human evolution and a growing public health concern in contemporary human populations.

## Author Contributions

AK and BZ conceived of the idea for this manuscript and wrote the manuscript.

### Conflict of Interest

The authors declare that the research was conducted in the absence of any commercial or financial relationships that could be construed as a potential conflict of interest.
